# Alzheimer’s disease – the journey of a healthy brain into organ failure

**DOI:** 10.1186/s13024-022-00523-1

**Published:** 2022-03-05

**Authors:** Todd E. Golde

**Affiliations:** Departments of Neuroscience and Neurology, Evelyn F. and William L. McKnight Brain Institute, Norman Fixel Institute for Neurological Diseases, Center for Translational Research in Neurodegenerative Disease, University of Florida, Gainesville, Fl USA

**Keywords:** Alzheimer’s Disease, Proteinopathy, Neurodegeneration, Amyloid, Tau, Organ failure, Risk factors

## Abstract

As the most common dementia, Alzheimer’s disease (AD) exacts an immense personal, societal, and economic toll. AD was first described at the neuropathological level in the early 1900s. Today, we have mechanistic insight into select aspects of AD pathogenesis and have the ability to clinically detect and diagnose AD and underlying AD pathologies in living patients. These insights demonstrate that AD is a complex, insidious, degenerative proteinopathy triggered by Aβ aggregate formation. Over time Aβ pathology drives neurofibrillary tangle (NFT) pathology, dysfunction of virtually all cell types in the brain, and ultimately, overt neurodegeneration. Yet, large gaps in our knowledge of AD pathophysiology and huge unmet medical need remain. Though we largely conceptualize AD as a disease of aging, heritable and non-heritable factors impact brain physiology, either continuously or at specific time points during the lifespan, and thereby alter risk for devolvement of AD. Herein, I describe the lifelong journey of a healthy brain from birth to death with AD, while acknowledging the many knowledge gaps that remain regarding our understanding of AD pathogenesis. To ensure the current lexicon surrounding AD changes from inevitable, incurable, and poorly manageable to a lexicon of preventable, curable, and manageable we must address these knowledge gaps, develop therapies that have a bigger impact on clinical symptoms or progression of disease and use these interventions at the appropriate stage of disease.

## Background: the nosology and epidemiology of AD

Nosology is the term used to describe the naming or classification of disease. The seminal contributions made by Drs. Fischer and Alzheimer still provide the underpinnings for the current nosology of Alzheimer’s disease (AD) [1–4]. Both scientists documented the core pathological features of the postmortem AD brain – senile plaques, neurofibrillary tangles (NFTs), neuronal loss and gliosis. The presence of these changes in the cortex and hippocampus upon neuropathological examination was associated with a progressive amnestic dementia and decline in other higher order cognitive functions prior to death. Indeed, AD could equally be called Fischer’s disease or perhaps plaque and tangle dementia, but as elegantly discussed by others, political factors and scientific rivalries led to naming this form of dementia AD [5–8].

AD is the most common form of dementia among the elderly, accounting for ~ 70% of all dementias in those over 60 [9]. Currently, worldwide, ~ 40 million individuals are affected by AD, a number that will grow to over 100 million by 2050. Estimates of AD prevalence vary based on the population studied and criteria used to clinically define the disease, but all studies show increased prevalence with increasing age. For example, in the US a 2010 study showed a prevalence of 3% among those 65–74, 17% of those age 75–84 and 32% for those 85 years and older [9, 10]. Thus, with increased life span comes an increased risk for developing AD.

AD typically manifests with short-term semantic memory problems but progresses to alter many higher order cognitive functions. Other cognitive and behavioral disturbances are common, but variable, features, which can present during disease progression. These features include, but are not limited to depression, anxiety, irritability, apathy, euphoria, disinhibition, psychosis, agitation, aggression, aberrant motor activities, sleep disturbance, and eating disorders. On average individuals live for ~ 7 years with AD, but some progress more rapidly and others much more slowly [9]. Ultimately, those affected with AD die because of complications of the disease often related to infections such as pneumonia. Given the slow progression and its prevalence, AD extracts a huge personal, societal, and economic toll – a toll that will dramatically increase in the coming decades if highly effective interventions are not developed. Indeed, monetization of the impact of AD indicates that it costs the US ~ $300 billion dollars a year [9].

Our understanding of AD and other forms of dementia has evolved significantly since its initial neuropathological description in the early 1900s. In the 1980s, AD entered the “molecular age” when a small neuropeptide called the amyloid β protein (Aβ) was found to be the key fibrillar component of senile plaques [11–14] and the microtubule binding protein tau to be the key fibrillar component of NFTs [15, 16]. We also now recognize i) that based on clinical symptoms many forms of dementia can be difficult to distinguish from AD and ii) the long prodromal phases of AD reflect an underlying pathological disease process long before overt clinical symptoms appear [17, 18]. Clinical neuropathological correlations demonstrate that AD does not always present with the classic amnestic syndrome characterized by early defects in episodic memory (reviewed in [19]). Pathological subtypes of AD such as hippocampal sparing AD and posterior cortical atrophy can be associated with clinical symptoms more commonly linked to frontal temporal dementia or prion disease. Moreover, “pure” AD defined by only plaques and tangle pathology is rare in those over 80. Other pathologies such as TDP-43 inclusions, hippocampal sclerosis, α-synuclein in Lewy Bodies and Lewy Neurites and vascular changes are often, but variably, present [19].

The advances that have helped us refine our nosology of AD are not always reflected in our interpretation of prior data. Past studies of dementia in the elderly that have relied on clinical diagnosis are confounded by the classification of non-AD dementia as AD and, if assessing cohorts in the very early stages of dementia or mild cognitive impairment (MCI), often included many individuals without overt neuropathology. Further, a significant percentage of elderly controls will have varying degrees of underlying AD pathologies [20–24]. Indeed, confounds are caused both by misclassification i) of non-AD dementias as AD and ii) and non-demented controls who are in some preclinical stage of AD. These confounds should be considered in our interpretation of prior studies that rely solely on the clinical diagnosis of AD or MCI. Armed with both imaging and emerging blood-based biomarkers, we now can conduct rigorous clinical studies in living humans by more accurately diagnosing AD and AD pathologies even in those without overt symptoms.

### Towards a comprehensive theory of AD

**Fig. 1:**
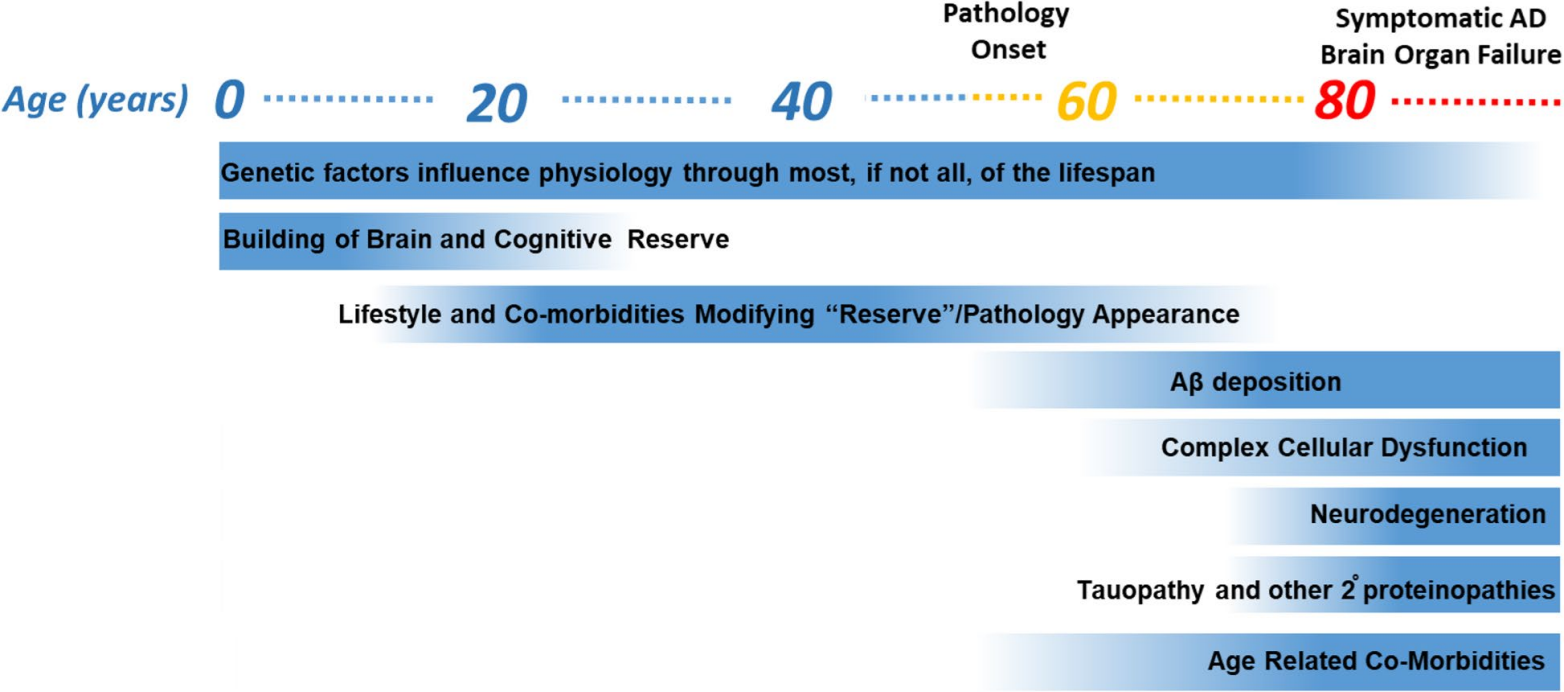
The lifelong journey of a healthy brain into AD and brain organ failure. The factors that contribute to the development, and alter risk for, AD are depicted below a hypothetical lifespan of someone who gets symptomatic AD at 80 years of age. The blue coloring indicates the time and strength of those factors that are active or emerge variably during the lifespan. Pathology onset begins some 25–30 years before symptoms emerge and shows a characteristic sequence of changes beginning with Aβ deposition followed by cellular dysfunction, tau pathology and neurodegeneration. Age related co-morbidities can have increasing impact on disease course in late life. Brain and cognitive reserve can alter symptom emergence and progression without altering underlying pathology

Any comprehensive theory of AD must be able to account for many disparate observations and also be able to distinguish between the relatively rare evidence that points to causality versus the common evidence that provides simple association with disease. To have face validity, a theory must be able to account for both more settled scientific aspects of the disease and provide plausible insights into areas of the pathological cascade that are more controversial. Here, I provide a modern rigorous conceptualization of AD conveyed as a hypothetical lifelong journey of human brain from a healthy state into organ failure (Fig. 1). Though AD is conceptualized as a disease of aging, and advancing age is irrefutably the major demographic risk factor for AD, processes underlying AD are present in the developing brain and persist throughout the life span. These data build off the long-standing amyloid cascade hypothesis (ACH) [25, 26], but acknowledge that the linear cascade proposed in the ACH is far more complex than originally thought [27].

### Genes set the stage

**Fig. 2:**
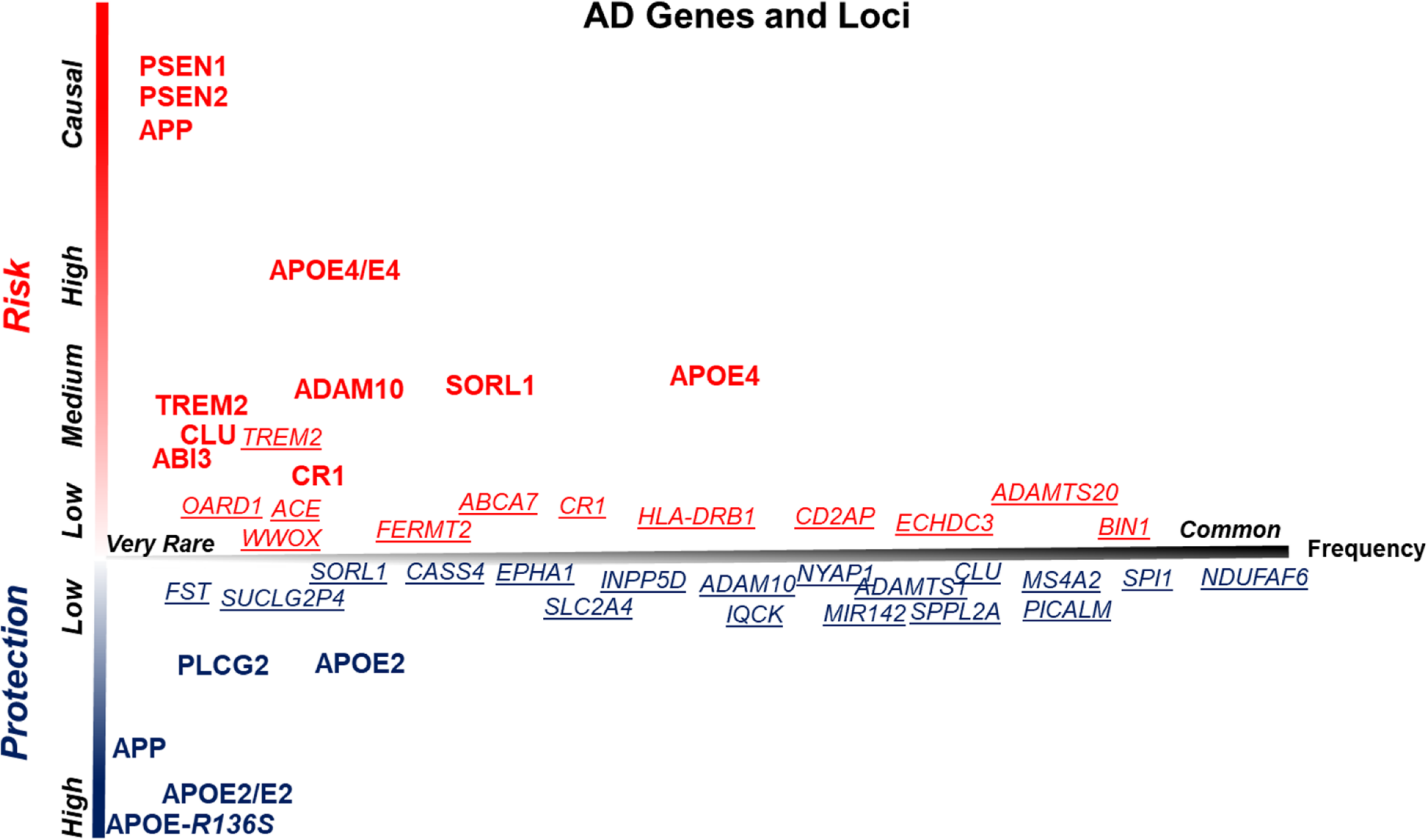
AD Genes and Loci associated with AD risk. AD genes are indicated in bold text whereas loci implicated by genome wide association studies (GWAS) are underlined. Y-axis indicates bidirectional risk, and the x-axis indicates the frequency of these mutations of variants. Note that several AD genes have both harmful and protective mutations or variants. For most loci implicated by GWAS, the genes and functional variants that are driving the association remain unknown. GWAS loci are derived from Kunkle et al. Nature Genetics 2019. 51(3):414–430 [32]

AD has a strong heritable component [9, 28–30]. Epidemiologic studies, including monozygotic twin studies, show that the heritability of AD is at least 50%. Having a parent or sibling with AD increases risk, and those with more than one first-degree relative are at even higher risk. Thus, genetics plays a major role in risk for developing AD. The field has now catalogued, at least in Caucasians of European decent, i) genetic alterations that deterministically cause familial AD, ii) the more common genetic variants that alter risk for AD, and iii) the relatively rare variants with MAF of ~ 0.01 that reproducibly associate with altered AD risk (Fig. 2) [31–42]. Rare variants with minor allele frequency (MAF) less than 0.01 that alter risk for AD are still being identified, though the low frequency of these variants in the population often means that validation of the variant with AD risk will take some time. Well-powered, genetic, biomarker and autopsy studies will be needed to confirm the genetic association and to show that the association is truly linked to biomarker or pathologically verified AD. In the current genome wide association studies (GWAS) that rely on a clinical diagnosis of AD, potential inclusion of non-AD dementing syndromes might mean that some risk loci identified are not really AD loci but loci for other forms of dementia. In this light, a very recent AD GWAS of over a million individuals identifies both *GRN* and *TM106b* as AD risk genes, whether this represents true genetic pleiotropy or simply inclusion of sufficient FTD-*GRN* cases misclassified as AD will require further study [43].

Extremely rare variants in the amyloid β precursor protein (*APP*) or Presenilin 1 (*PSEN1*) or 2 (*PSEN2*) genes can cause autosomal dominant AD, which usually presents as early-onset AD (EOAD) [44–58]. Symptoms usually manifest within these families between the ages of 40–60. Of great relevance, a rare variant in the *APP* gene has been shown to protect from AD in Icelanders [59]. In contrast to these rare AD-linked mutations in *APP* and *PSEN1/2*, the *APOE* gene confers a large population attributable risk for AD. The *APOE4* allele is associated with increased risk and the *APOE2* allele protection from AD [40, 41]. Both *APOE2* and *APOE4* alleles alter risk in dose dependent fashion; the presence of one allele confers a moderate risk or protection but the presence of two alleles confers even stronger risk or protection. Indeed, given that AD is a common disorder, the ~ tenfold risk attributable to a homozygous *E4/E4* genotype verges on causality, whereas the even rarer *E2/E2* genotype results in almost complete protection [60, 61]. More recently homozygosity of a rare variant in *APOE*, referred to as the Christchurch variant (R136S) was associated with long lasting protection from symptoms of AD in a single-carrier of the early-onset familial AD (EOFAD)-linked *PSEN1 E280G* variant [62]. In contrast a heterozygous carrier of this same variant developed clinical symptoms of early AD at 53 years and died at 72 years of age [63]. Postmortem analysis showed classic features of Alzheimer’s disease. Other rare variants in *APOE* also confer protection, though such findings need broader replication [64]. Undeniably, such studies highlight the challenges of making broad inferences about causality or protection from what are essentially case-reports.

### The biologic basis of genetic risk – the life-long impact of genetic variance

**Fig. 3:**
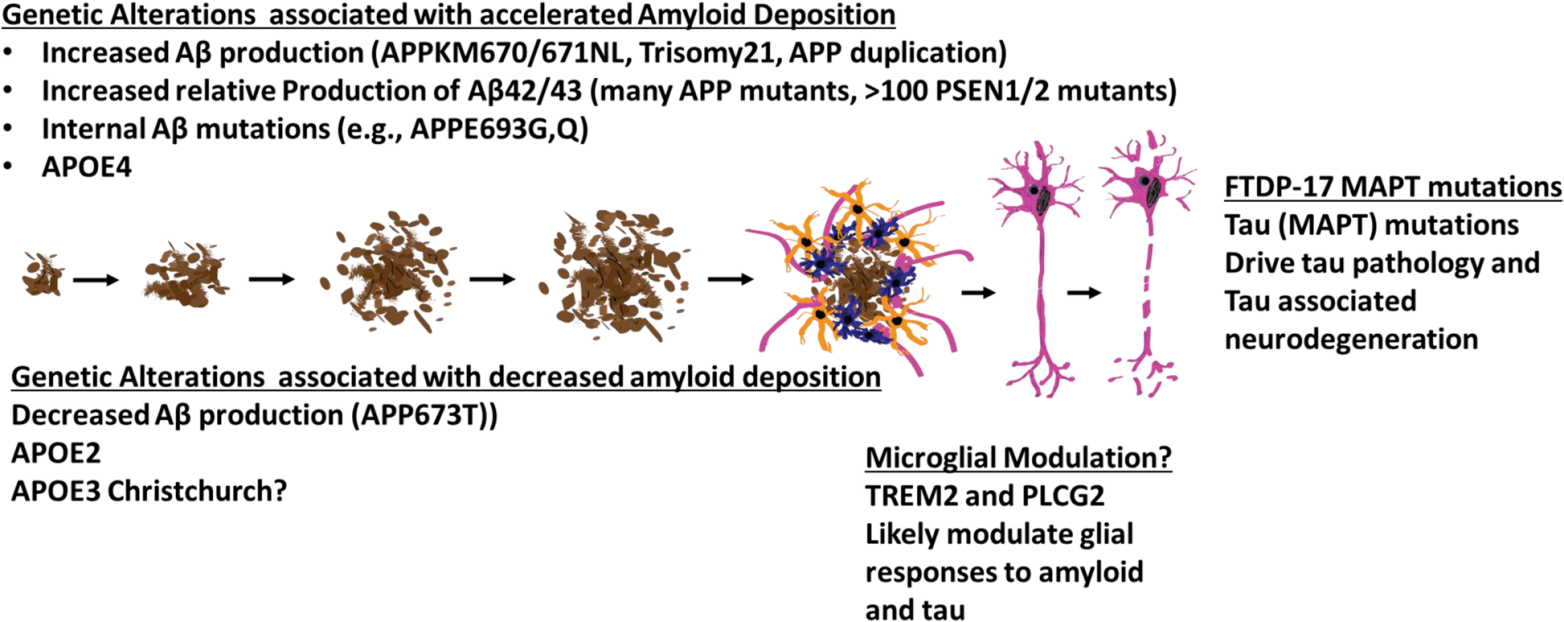
Functional impact of genes linked to AD or risk for AD. Pivotal support for the ACH comes from genetic pathological, biomarker and experimental studies that link genes that cause EOFAD or alter risk for AD to Aβ aggregation and accumulation. Mutants and variants that increase risk alter Aβ in a manner that promotes deposition as amyloid. Conversely other variants appear to decrease the likelihood of Aβ aggregation and accumulation. AD genes, enriched in microglial cells, may modulate Aβ deposition, responses to aggregated Aβ or tau pathology, or both. Mutations in tau associated with FTD reinforce the notion that tau aggregation and accumulation is an important feature of AD

In the absence of effective proven disease modifying therapies or faithful animal models that fully recapitulate the human disease, genetic studies serve as the only truly validated guideposts for inferring causality (Fig. 2). Study of these rare genetic forms of AD have provided the framework for the ACH and our current understanding of AD at least with respect to the initial causal triggers of disease [26, 65]. Biomarker, modeling, biophysical and pathological studies all show that mutations in *APP* or *PSEN1/2* associated with early onset Familial AD (EOFAD) result in changes to APP processing or Aβ itself, in a way that promotes Aβ aggregation. (Fig. 3). They do so in three distinct ways: i) increasing total Aβ production [45, 66–69], ii) increasing the relative production of long Aβ peptides (typically Aβ42 but sometimes Aβ43) [70–74] or iii) altering the sequence of Aβ itself in a way that promotes its aggregation [44, 75–77]. In contrast, the protective variant in APP found in Iceland is associated with decreased Aβ production [59], and confers life-long protection from AD and cognitive decline. These findings serve as the fundamental observation that supports the ACH, which in its simplest form posits that Aβ aggregation, as amyloid, is the initiating event in AD.

Notably, these mutations appear to cause life-long changes in Aβ production or clearance. However, the accumulation of Aβ is still quite gradual typically preceding onset of symptoms by at least 20–30 years [18, 78]. Timing of accumulation appears to be primarily determined by both the total Aβ level, the relative level of Aβ42 (or in some cases both Aβ42 and Aβ43) and *APOE* genotype [79]. Both autopsy and imaging studies show that *APOE4* leads to earlier onset of Aβ deposition and typically results in higher levels of Aβ deposited, whereas *APOE2* delays onset of deposition [61, 80–84]. This difference translates into the clinical setting where *APOE4* carriers typically show increased incidence of AD and an early onset of symptoms compared to those with *APOE3* alleles whereas *APOE2* carriers show reduced incidence of AD and delayed onset [85–92].

Aβ is produced from APP constitutively through the combined action of the aspartyl proteases β-secretase (BACE1) and γ-secretase [93–98]. PSEN1/2 serve as the catalytic component of the multi-subunit γ-secretase complex [99–106]. This sequential processing produces a complex set of Aβ peptides with variable amino and carboxyl termini [107]. Thus, Aβ is best referred to as a ~ 4 kDa peptide [108]. In vitro “Long” Aβx-42(43) species aggregate much more rapidly than shorter Aβ peptides (e.g., Aβx-38, x-40) [109–111], and this is reflected by the early and preferential accumulation of Aβx-42 in humans [112, 113]. In the absence of mutations in the Aβ peptide sequence itself, the longer Aβ species appear to be required for deposition of Aβ in vivo [114, 115]. Curiously, in humans, most mutations within the Aβ peptide sequence that alter its aggregation properties variably cause AD, severe congophilic amyloid angiopathy (CAA), or some combination of the two. CAA caused by Aβ typically shows Aβ40 preferentially accumulating in the leptomeningeal arteries and cortical arterioles [44, 75, 112, 116, 117]. CAA is the predominant feature in the autosomal dominant disorder Hereditary Cerebral Hemorrhage with Amyloidosis Dutch type (HCHWA-D) linked to mutations at position 693 of APP, which clinically presents with cortical hemorrhagic stroke. However, amyloid deposition in the brain parenchyma is present and likely contributes to disease progression in HCHWA-D. CAA due to Aβ is also a common feature of AD, and small cortical hemorrhages associated with CAA may contribute to the cognitive phenotype in AD. In the general population, large bleeds due to CAA that contribute significantly to functional decline are rare, but do occur. These bleeds can often confound clinical diagnoses in the absence of imaging studies. However, though CAA may play some role in the overall phenotype, it still is important to note that fulminant AD can occur without appreciable CAA [117].

There is compelling data from model systems that select EOAD-linked *APP* and *PSEN* mutant may alter cellular functions such as endosomal, autophagic and lysosomal trafficking [118–120]. These effects in some cases appear to be independent of effects on APP processing and subsequent alterations in Aβ [121]. There are other GWAS loci implicated in endosomal trafficking [32]. However, direct links between these alterations associated with variants in *APP*, *PSEN*, and GWAS loci and AD pathophysiology remain somewhat uncertain. More generally, there is limited data regarding the link between AD genetic risk loci identified in GWAS and the alteration in function that contributes to AD pathogenesis. Imputation of biological function of genes implicated by GWAS do point to immune system, lipid metabolism, tau-binding proteins, and APP metabolism [31, 122]. However, for most associations imputations to biological function remain speculative. As the risk associated with most GWAS loci is small, it may prove very challenging to home in on the biological basis for the genetic risk.

As the most abundant lipoprotein in the brain, APOE is linked to numerous biological functions related to its role in lipid transport [123, 124]. However, APOE is intimately linked to Aβ pathophysiology in an isoform dependent fashion that is consistent with the isoform-associated impact on AD risk. In humans, APOE co-deposits with Aβ in cores of plaques. In autopsy studies, the *APOE4* allele is associated with increased parenchymal and vascular amyloid deposition, whereas the *APOE2* allele is associated with less amyloid deposition. In amyloid PET ligand imaging studies, *APOE4* is associated with increased amyloid ligand binding and an earlier average age of onset of detectable ligand binding, whereas *APOE2* showed the opposite effect, delaying average age of onset and reducing amount of ligand accumulation [123]. Differences in biological impact of APOE isoforms has also been noted with respect to lipid metabolism, immune cell function, cardiovascular health, and even to tauopathy in mouse models [123–126]. However, it remains unclear whether these are major contributors to the isoform-dependent alterations in risk for AD conferred by *APOE*. *APOE* and other genetic alterations linked to AD have early life effects that are related to their normal biological functions. For example, *APOE* genotype has been linked to hippocampal volume and myelination alterations in infants and pediatric populations [125, 127]. However, these subtle alterations do not impart major effects on cognitive abilities or have other effects on brain and cognitive phenotypes.

Both immune loci implicated by GWAS and rare variants in *TREM2, ABI3* and *PLCG2* are thought to alter AD risk by influencing microglial or more broadly immune function [33, 38, 58]. Although far from settled science, study of the functional variants in TREM2 and PLCG2 suggest that increased microglial activation mediated by these proteins is protective from AD [128–134]. As discussed later, the impact of immune system on AD pathogenesis likely occurs later in life as the proteinopathies emerge. Of note, Dr. Fischer presciently, postulated that plaques might represent some sort of pathogen and that the glial response might contribute to disease progression. Indeed, the notion that infection, such as herpes virus, may play a causal role in AD, continues to find some traction the field. However, this infectious origin of AD hypothesis remains controversial and is largely based on less than convincing epidemiologic association studies [135]. Indeed, it is more widely accepted that Aβ aggregates are danger-associated molecular patterns that potently activate the immune system similar to an exogenous foreign pathogen [130, 136].

### Early life brain development, educational attainment and risk for AD

Both educational attainment and intelligence are among select factors reproducibly associated with altered risk for late life dementia and AD [10] (Fig. 1). These observations have provided foundational support for the concept of cognitive and brain reserve [137, 138]. As with most epidemiologic associations, detailed mechanistic insight into the biology underlying the risk is lacking, but the general concepts are quite simple. If one has a more “fit” brain that is nurtured during development and early life then it is capable of withstanding more damage later in life. Alternatively, one could propose early brain development creates a more fit brain that actually alters the risk for development of AD pathologies later in life. Current data more strongly supports the notion that these early life factors do not alter the presence of pathology, but the ability to retain cognitive function despite damage to the brain [137, 138]. The biological factors that underlie reserve are speculative. The field has conceptualized reserve into two main categories: brain reserve, which refers to brain structure changes that may increase tolerance to pathology, and cognitive reserve, which refers to individual differences in how tasks are performed that might enable some people to be more resilient to brain changes than others [138]. Developmental processes that lead to a more resilient brain structure and inherent attributes that could relate to synaptic and neuronal plasticity could contribute to both forms of reserve. As intelligence is highly heritable, it is not surprising that negative correlations between genetic variants that are associated with higher measures of intelligence and AD have been reported. However, there is no tight functional link between variants that associate with intelligence levels and those that associate with AD [139].

### Lifestyle and co-morbidities influence cognitive trajectories

Numerous epidemiologic studies have now shown that a healthy diet, physical exercise, reduction of cardiovascular and metabolic disease risk factors and control of hypertension are associated, at the population level, with modest reductions in risk for dementia in later life [140–149]. The biological factors that underpin these associations are not well established and are likely to be highly complex and heterogeneous in nature. Here it is important to distinguish between the broader umbrella of late onset dementia and cognitive impairment that is assessed in these studies versus AD dementia. These “lifestyle” factors clearly impact cardiovascular and vascular health, and many believe that beneficial effects of these various factors are mediated through improved vascular health. Thus, the associations may be more reflective of the co-morbid conditions that contribute to brain reserve or fitness, rather than directly impacting AD pathophysiology (Fig. 1). Of course, there are studies in model systems that suggest that factors such as diet and diabetes could more directly impact AD pathologies, but these have not been widely replicated and the effects sizes of these manipulations are relatively modest.

Whether viewing these associations from an individual or public health perspective, it is sensible to encourage individuals who are worried about dementia in later life to adopt lifestyle changes that evidence suggests should support future protection from dementia. Indeed, beneficial lifestyle changes have many overall health benefits. The challenge here is one of implementation. How do we get individuals to adopt such lifestyle changes? Efforts to understand the factors that mediate these beneficial effects, and the mechanisms through which lifestyle changes alter physiology to promote healthy brain aging, are laudable. However, it is likely that the underlying biologic impact of these lifestyle factors is multilayered and multifactorial.

Other factors such as stress, depression, sleep alterations and traumatic brain injury (TBI) have been associated with an increased risk for dementia later in life [150–156]. Whether these are truly risk factors for AD remain somewhat uncertain, as these studies rely solely on a clinical diagnosis of “AD”, which as noted above cannot precisely distinguish AD from other forms of dementia. Indeed, TBI is much more consistently associated with a post-mortem diagnosis of chronic traumatic encephalopathy (CTE) [157–160]. CTE is primarily considered a tauopathy and has distinct spatiotemporal distribution of tau compared to AD. However, for stress there is strong preclinical biology indicating that dysfunction of the hypothalamic pituitary axis may accelerate both amyloid and tau pathologies and possibly even hippocampal neurodegeneration [150]. Other behavioral alterations such as disrupted sleep have also been demonstrated to alter Aβ production, accumulation, efflux from the brain or some combination of these effects [161, 162]. Thus, one could envision how depression and other chronic stress conditions that often disrupt sleep could contribute to risk for future AD. Nevertheless, as the pathophysiology of AD begins long before symptoms appear, another concern with many of these retrospective epidemiologic studies is whether the associations reflect a true prodromal risk or are actually early subtle signs of pathological changes in the brain. In any case, non‐genetic factors may be more amenable to intervention than heritable aspects of AD, especially if such interventions are non-pharmacologic in nature and safe. Therefore, even though it is challenging, it is important to establish more definitively what non‐genetic factors contribute to AD risk and the biological basis for that risk.

### The long, clinically silent, phase of AD pathology development

Over the last 20 years, there have been remarkable advances in our ability to image AD pathologies and structural changes in the brains of living patients, and in many cases to do so longitudinally. PET ligands can now detect amyloid and tau pathologies [163–170]. Structural magnetic resonance imaging (MRI) imaging can evaluate brain volumetric changes and alterations in brain connectivity in AD [165, 171]. More recently, advances in blood biomarkers that accurately predict underlying AD pathologies, even in the absence of symptoms, portends a new era where simple laboratory tests may provide substantive insight into the status of the brain with respect to underlying AD pathologies [172–181]. All of these blood-based biomarkers were initially developed and validated in studies using cerebrospinal fluid (CSF), but technological advances enabled current serum or plasma assays [18, 165]. Indeed, decreased Aβ42:40 levels and increases in select phosphorylated tau epitopes are associated with amyloid PET ligand positivity even in asymptomatic stages. Neurofilament light chain (NFL) elevations correlate with neurodegenerative findings on MRI and in some cases progression of symptoms or rate of progression to symptoms. Glial fibrillary acid protein (GFAP) increases are associated with astrogliosis. NFL and GFAP are not specific for AD, but in the setting of evidence for amyloid deposition, they add vital information about the stage of disease.

These biomarker studies now consistently show that twenty or more years before one develops symptoms of AD, Aβ begins to accumulate as amyloid in the brain [182–184] (Fig. 1). For many years, this gradual deposition appears, at least at the level of cognitive function, to have relatively little impact. Though some cognitive “stress tests” may be able to detect subtle changes in cognition in these individuals, exactly when those changes occur and how consistent they are remains under investigation [185]. Indeed, it is only after a decade or more of ongoing progressive accumulation of Aβ that signs of neurodegeneration appear, and along with these signs, evidence of impaired brain function. However, even at this stage of disease, functional and cognitive changes are still not enough to fall out of the normal range. Tau pathology of sufficient magnitude to be detected by PET imaging appears to begin coincident with the onset of neurodegeneration detected by volumetric brain loss on structural MRI imaging. The timing of glial, vascular and synaptic changes within this framework remains less clear, but most studies would suggest that such changes are roughly coincident with the transition from an amyloid only state to an amyloid plus neurodegeneration state.

**Fig. 4:**
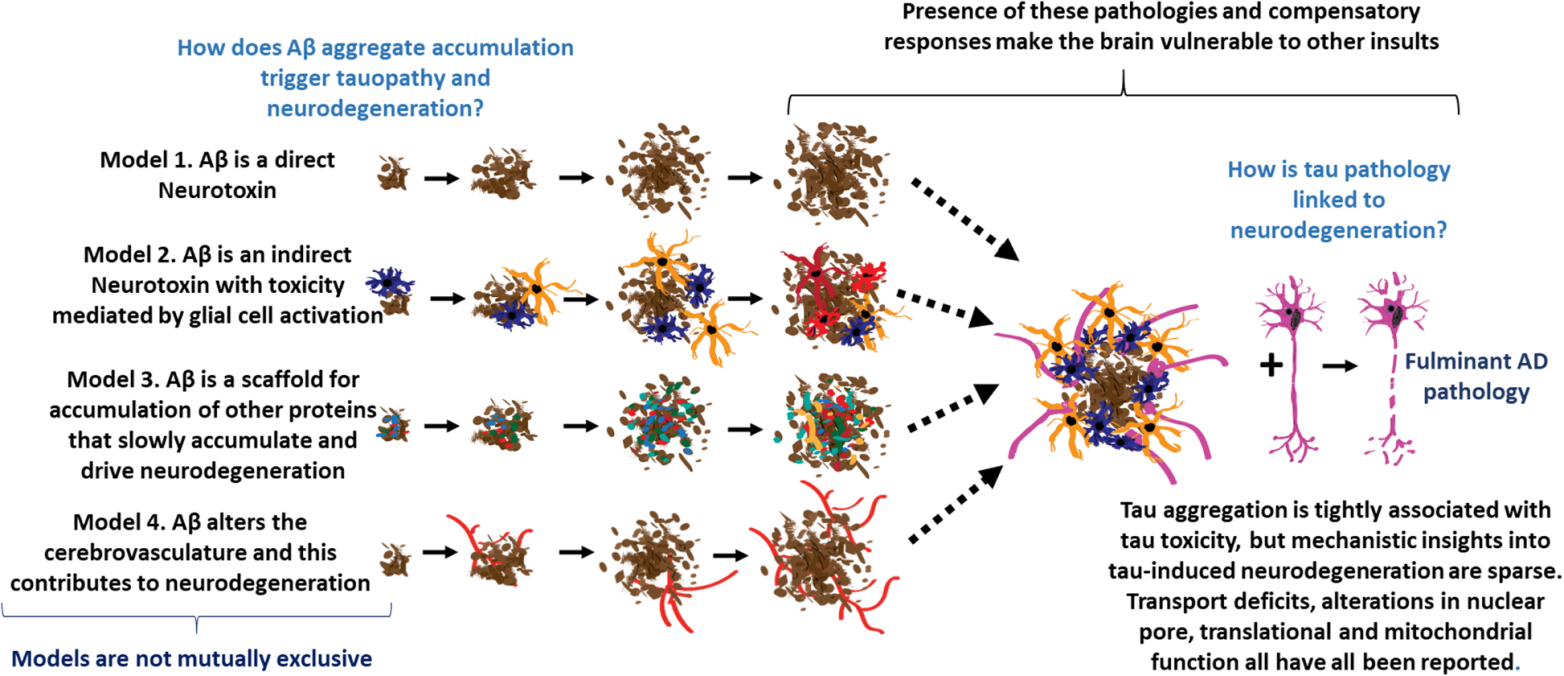
Enigmatic aspects of the ACH. This schematic illustrates some of the gaps in our knowledge regarding certain aspects of the ACH. Uncertainty remains about how Aβ aggregate accumulation drives downstream changes. Similarly, there is uncertainty regarding how tau accumulation leads to neurodegeneration. It is likely that the mechanism that link Aβ and tau together and to neurodegeneration are complex. One concept that is not commonly discussed is that the presence of either amyloid or tau pathology and both pathological and compensatory response may make the brain more vulnerable to additional insults. Such vulnerability might explain why clinical progression is so variable, and how co-morbidities may impact the clinical phase of disease

Cross-sectional studies of postmortem human brains enabled scientists to infer the pathological sequencing now seen in virtually every presentation of biomarker studies of AD. Indeed, Braak and Thal staging of tangle and amyloid pathology, respectively, were well established staging frameworks for AD based on post-mortem studies [186, 187]. However, until modern imaging and biomarker studies enabled robust studies in living humans, these postmortem findings were interpreted in very different ways. Some argued that the presence of Aβ in individuals who died with normal cognition meant that Aβ accumulation did not play a causal role in the disease, and could even be protective [188–190]. Given data in hand in the 1980s, this postulate was reasonable. However, as the biological underpinnings of genetic forms of AD emerged in the 1990s and consistently pointed to Aβ accumulation as an early triggering event, the concept that Aβ deposition was benign became less tenable. Notably, the lack of definitive mechanistic insight into how Aβ accumulation triggers the neurodegenerative phase of the disease strongly suggest that though amyloid is necessary to trigger AD it is not sufficient (Fig. 4). It remains plausible that plaque formation is an acutely adaptive response that is initially protective but over time turns toxic. There are certainly many precedents for acutely adaptive biological responses in humans that later result in toxicities.

Neurodegenerative changes occur during this prodromal silent phase of disease. However, there is individual variability in time between onset of degeneration and symptomatology that triggers a diagnosis of mild cognitive impairment or dementia [165]. Studies in EOFAD as well as some other longitudinal cohort studies do show that as signs of neurodegeneration emerge there are associated changes in cognitive function. In most cases, these changes would not be sufficient to recognize that the individual has some sort of cognitive impairment. Rather, they simply demonstrate a change from an individual’s baseline cognitive status. Again, brain and cognitive reserve likely play a major role in determining how much damage an individual brain can tolerate before symptoms become overt. Individuals with high reserve may tolerate much higher levels of pathology and neurodegeneration before showing symptoms, and those with lower reserve may show symptoms sooner [137].

### Symptomatic phase of disease – progression from early to late brain organ failure

Once neurodegeneration begins, there is variable, yet inexorable, progression towards symptomatology that is clinically recognized as abnormal. Within the brain, this is attributed to ongoing neuronal and synaptic loss, spread of tau pathology, and alterations in the cellular function of non-neuronal cells [165]. As this pathology advances, so do the symptoms. Subjective memory complaints may be a very early sign of something wrong, but have indeterminate predictive value. Often the spouse or caregiver notes the individual has shown some cognitive alterations for years before presenting to a clinician. Consistent or increasing concern about cognition may lead to clinical assessments and diagnosis of mild cognitive impairment (MCI). An individual with MCI often complains of forgetfulness, losing their train of thought, having difficulty with decision making and having trouble navigating their environment. A spouse or caregiver may report that they are more impulsive and showing poor judgment. Signs and symptom of depression, irritability, anxiety, and apathy may also be present. Of note, not all individuals with MCI, as diagnosed solely by clinical criteria, progress to dementia. However, semantic memory deficits in MCI are highly predictive of a future dementia. Further, MCI with biomarker evidence for amyloid deposition is also highly predictive of future AD [165, 191, 192].

Although MCI is recognized as the earliest clinically distinguishable stage of potential incipient dementia, it is not early from a pathological perspective. All of the underlying pathologies have been present for many years before someone is diagnosed with MCI, and structural changes and neurodegeneration are already present. Aβ is likely to have been present and even plateaued in levels for over a decade (Fig. 1). Thus, even the earliest symptomatic stages of AD represent a long-standing pathology and early brain organ failure [165]. As MCI progresses to overt dementia, symptoms become more pronounced, with memory and executive function deficits becoming prominent. Individuals become less and less capable of retaining new information and social judgment becomes impaired to the point where function outside of the home is difficult. Assistance for personal care and hygiene is needed in the later stages of the disease and the individual becomes highly dependent on others. Agitation, irritability and apathy all may be present. Eventually an individual with late stage AD becomes debilitated and often bedridden. Often death with AD is attributable to an infection, as many develop and die from pneumonia.

Symptoms in AD inexorably progress but that progression is not always linear. Co-morbidities and additional insults can cause more step-wise functional loses, and sometimes more sudden changes in cognition occur without apparent cause. The importance of such co-morbidities in AD progression is likely underappreciated. Indeed, even small strokes in the setting of underlying AD pathology may appear to have exaggerated functional effects [193, 194]. Further, transient and treatable infections such as urinary tract infections may acutely alter cognitive function and accelerate long-term functional decline. The impact of co-morbid conditions is especially apparent in individuals with dementia over 80, who based on postmortem studies rarely have pure AD. Instead, these brains often harbor multiple pathological changes that are associated with various other forms dementia. Vascular changes in the elderly are especially important contributors, but are not easy to assess at autopsy.

Postmortem examination of brains with AD or MCI of the AD type, show extensive damage. On average there is more amyloid and tau pathology in those individuals who die with more advanced symptoms, but even recent transcriptomic and proteomic studies reveal thousands of changes in gene expression and protein levels indicative of a complex long-standing degenerative process that also certainly represent compensatory responses [195–197]. Symptomatic phases of AD truly represent brain organ failure.

### Compelling, direct support for the ACH hypothesis

As described above, compelling genetic, pathological, modeling and human biomarker data support a contemporary version of the ACH as a framework for understanding the progression of a healthy brain into brain organ failure due to AD pathology. Slow, progressive accumulation of Aβ aggregates triggers AD by initiating a complex pathological cascade that accelerates tau pathology, alters glial cell function and neurodegeneration and ultimately leads to clinical dementia. Genetics and lifestyle may interact to influence risk and the associated pathologic processes. Cognitive and brain reserve and comorbidities that occur with increasing frequency during aging variably contribute to alter the clinical manifestations of the disease, as well protection from it.

Much of the most critical data that causally implicated Aβ aggregation in AD comes from the study of EOFAD and modeling the impact of the genetic alterations found in FAD. Human biomarker studies, as well as clinical and pathological studies show, with few exceptions, that the natural history of EOFAD is very, very similar to the natural history of more typical late onset AD [198, 199]. Thus, there is a strong foundation for extrapolating insights based on EOFAD to late onset AD. Moreover, studies of genetic variants that influence risk for late onset AD both in humans and in models demonstrate that the impact of the genetic variant is consistent with postulates of the ACH. Of course, it must also be acknowledged that late onset AD occurs in the setting of advancing age, and that the associated comorbidities and other factors that result in physiological effects of aging may alter the clinical course of AD in important ways.

### The ACH is indirectly supported by the study and understanding of other neurodegenerative proteinopathies

Though the data described above that supports the ACH in AD is highly compelling, additional supporting evidence has emerged from the study of other neurodegenerative disorders. Both familial British and Danish dementias (FBD and FDD, respectively) are close phenocopies of AD, both pathologically and clinically [200–202]. In these disorders, small peptides (Abri or Adan), like Aβ, accumulate as extracellular amyloid in the brain. This accumulation is clinically associated with a progressive clinical dementia plus additional symptoms attributed to deposition in non-cortical structures. Neuropathological assessments show the non-Aβ amyloid plaques and CAA, NFTs, neuronal loss and gliosis. Further, there is strong evidence that other neurodegenerative diseases, ranging from polyglutamine disorders to many forms of ALS, Parkinson’s disease, and frontotemporal dementias (FTD), are essentially proteinopathies. Like AD, these diseases appear to be triggered by the accumulation of aggregated proteins in alternatively folded beta-pleated sheet amyloid or amyloid-like structures, either within or outside the cell [203, 204]. Indeed, crucial support for tau aggregation as a likely driver of neurodegeneration in AD comes from studies of FTD that is caused by mutations in the tau (FTDP-17 *MAPT*) [205, 206]. This form of FTD is a “NFT-only” dementia, and modeling studies show that tau mutations drive neurodegeneration and tau aggregation in the absence of amyloid. Finally, Aβ amyloid deposition in AD has intriguing similarities to peripheral amyloid diseases. Indeed, APOE, serum amyloid P component and heparan sulfate proteoglycan (HSPG) co-accumulation are common features of amyloid deposition in both AD and peripheral amyloidoses [207, 208]. Further in many peripheral amyloid disorders immune dysfunction especially of monocytes is a common feature.

### Critiques of the ACH

Each failure of an Aβ-targeting therapy in the clinic is followed by commentaries voicing skepticism regarding the ACH [209–213]. These commentaries typically ignore the vast majority of the aforementioned data that support the ACH. They also omit the key point that the trials have not tested the central postulate of the ACH that preventing Aβ accumulation will prevent AD. In 2003, I cautioned the field stating that testing anti-Aβ therapeutics in symptomatic disease had a low probability of clinic impact – too much damage has already been done [27]. To rephrase the analogy I used at that time: “True heart failure due to longstanding atherosclerotic disease and myocardial infarction is not reversed or significantly impacted by treatment with cholesterol lowering agents. So why should we expect Aβ-targeting therapies to have benefit in symptomatic AD?”. Other critiques of the ACH latch on to relatively isolated or restricted observations that are not completely consistent with early simplistic versions of the ACH, and inappropriately extrapolate from these isolated, and in some cases erroneous or poorly reproducible observations, to a statement that the ACH is wrong. Indeed, such observations do not invalidate the ACH, but rather suggests that the ACH in its original construction was oversimplified and did not fully capture the complex biological underpinnings of AD.

### Gaps in our understanding of the ACH

Perhaps the most critical aspect of ACH that is poorly understood is how Aβ pathology drives the downstream cellular dysfunction that leads to the neurodegenerative phase of the disease (Fig. 4). This central question needs to be answered in order to complete our understanding of AD, and provide a better framework for new therapeutic intervention. As discussed below, we do have clues as to how Aβ accumulation may drive downstream changes, but definitive insight is lacking.

Mainstream concepts have been that aggregates of Aβ are directly neurotoxic, trigger a toxic glial response, or both. Thousands of papers demonstrate the potential neurotoxicity of Aβ aggregates, and there have been extensive efforts to identify the singular most toxic species (e.g., oligomer, protofibril, dimer, specific fibrillar conformer) responsible for mediating the harmful effects of the Aβ on neurons [214–216]. Others have focused on where Aβ accumulates and suggested that intracellular Aβ may be particularly harmful [217]. However, multiple observations indicate that the link between Aβ accumulation and neurodegeneration is more complex. First, Aβ accumulates for 10–20 years before neurodegeneration is evident. So why is neurotoxicity not observed during this phase? Second, not all Aβ deposits elicit an equivalent harmful response. Aβ in diffuse plaques is not associated with overt pathology and is a common feature of the normal aged brain. In contrast, neuritic, fibrillar or compact plaques are associated with neurodegenerative changes, dystrophic neurites, tau pathology and a marked reactive gliosis [218]. So, what is the difference between these two forms of Aβ deposits? Third, in rodent Aβ deposition models, there is poor correlation between Aβ accumulation and neurodegeneration. Finally, much of the literature showing that select Aβ aggregates are “toxic” is based on application of exogenous Aβ aggregates to neurons in culture. Reports clearly demonstrating robust AD-like neurodegeneration induced by Aβ in vivo are far fewer and far less well reproduced.

Aβ aggregates are potent modifiers of glial biology and “reactive” astrocytes and microglia that surround plaques were described in the original pathological description of AD [219–224]. Genetic associations clearly link microglial genes to risk for AD. *TREM2, PLCG2,* and *ABI3* contain coding variants that influence microglial function [33]. Further numerous other GWAS loci harbor genes that are primarily expressed in microglia [32]. Notably, immune modulation in the brain can modulate both amyloid and tau pathologies, though there are now multiple examples of how the same immune manipulation moves these pathologies in opposite directions (reviewed in [130]). Further, immune signaling in the brain can drive neurodegeneration and synaptic alterations [130, 225, 226]. Thus, it is possible that Aβ aggregate mediated activation of glial cells and microglial in particular could trigger tau pathology, neurodegeneration and alter function of many different cells in the brain. However, we still have little definitive insight into both the how and when regarding the role of immune systems in mediating AD pathophysiology [130].

Multiple proteins with biological activity co-accumulate within Aβ deposits [227]. These include proteins that are genetically associated with AD such as APOE and CLU, as well as many other proteins that are known to play essential roles in brain signaling including but not limited to many HSPGs, signaling molecules such as midkine and pleiotrophin, and others such as A_1_ACT [228–232]. Modeling studies show that altered expression of these plaque associated proteins can alter both Aβ, gliosis, tauopathy and other pathological features such as dystrophic neurites. Thus, another emerging concept is that Aβ aggregate accumulation may not be sufficiently toxic to induce downstream neurodegeneration unless accompanied by accumulation of these proteins. Gradual, Aβ-dependent, accumulation of these proteins could overcome compensatory responses to trigger the neurodegenerative phase of AD, accounting for the long delay between onset of Aβ deposition and neurodegeneration in humans.

Aβ aggregates could alter the vasculature in AD a way that fosters additional pathologies. Clearly, CAA can have some impact on AD, but in many cases of fulminant AD there is little or no CAA pathology. Nevertheless, more subtle vascular changes could result in blood brain barrier disruption and that disruption, even if transient, could trigger downstream changes that promote neurodegeneration [233].

Of course, none of the possible mechanisms that *could* link Aβ aggregate accumulation to tau and neurodegeneration are exclusive; each may be operative either simultaneously or at various points in the disease (Fig. 4). Similarly, variations on these mechanisms could account for differential induction of other proteinopathies such as TDP-43 inclusion pathology or α-synuclein pathology. One of the more curious observations is that Aβ accumulation in mice and humans is associated with extracellular release of the amino terminus of tau truncated before the microtubule-binding microtubule binding domain [234–236]. Thus, the increase in these amino terminal species of tau is primarily a biomarker of amyloid pathology not tau pathology [237]. Such data suggest that there is some enigmatic link between amyloid and tau, but what that link in remains enigmatic. Speculatively, one wonders if Aβ accumulation induces the secreted form of tau; perhaps the cleavage of tau that produces this fragment might also generate a truncated species of tau that could be more prone to aggregation?

It is also important to note that we have similar gaps in our understanding of the relationship between tau, NFT and tau-associated neurodegeneration (Fig. 4). Tauopathy is more tightly associated with cognitive decline in AD, and study and modeling of FTDP-17 caused by mutations in tau reveals a tight link between tau aggregation and neurotoxicity, but we still lack detailed mechanistic insights into how tau dysfunction and aggregation damages brain cells [238–242].

Another important aspect to consider is that the presence of AD pathologies may make the brain more vulnerable to additional insults. Such vulnerability may help to explain why various co-morbidities can have appreciable impact on the clinical disease course (Fig. 1 and 4). Cells and circuits within the brain may be able to adapt and partially compensate for the presence of both amyloid and tau pathology, but those adaptations come at some cost -making the cells and circuits more vulnerable to another insult. This multi-hit concept of AD pathogenesis fits well with the typical protracted time-course, the imprecise correlations between symptoms and underlying pathologies, and real-world data that shows, for example, that even small strokes in individuals with early stage AD have major impact on disease course [193, 194].

### Beyond a neuron centric view of AD – embracing complexity

Perhaps one of the flaws in the way in which the experimental underpinnings of the ACH were explored was the aforementioned focus on trying to find a direct link between Aβ aggregation and toxic impact on neurons. As noted above, there are now multiple layers of evidence that there are pathophysiological alterations in all the cell types within the AD brain, and that these non-neuronal cells and factors produced by them contribute substantively to the disease cascade. A future challenge for the field will be to better understand the complex interplay among the various cell types, and model this interplay in systems that can provide reliable mechanistic insight. All of the pathology in AD is complex, and we will likely need to embrace that complexity in order to actually truly understand AD.

One area that deserves much more attention is the pathology referred to as dystrophic neurites (DNs). DNs that surround amyloid plaques are the one feature that distinguish AD from almost all other neurodegenerative disorders except for FBD and FDD [243–245]. DNs are swollen axonal structures filled with dysfunctional vesicles, many of which contain endosomal and lysosomal markers. Contact with the plaque may play a role in DN formation, but we have little knowledge of the precise mechanisms that catalyze their formation [243, 246–249]. Notably, a recent study suggests that DNs could serve as critical sites for the seeding of tau, and that tau aggregation in DNs precedes and catalyzes NFT formation [250].

More generally, a prominent area of research has been how tau pathology spreads [251, 252]. In model systems it is clear that prion-like, cell-to-cell spread of tau can occur. This mechanism is conceptually attractive, as it can explain the characteristic spread of tauopathy through the brain as AD progresses. However, it remains to be proven that this prion-like spread of tau actually occurs in human [203]. Nevertheless, the recent finding that tau pathology may be catalyzed within DNs is intriguing [250]. Other factors such as HSPGs that accumulate in DNs or even internalized Aβ aggregates could, theoretically, seed tau in DNs. Once seeded “in trans” tau pathology could than spread through a prion like mechanism, and essentially becoming independent of Aβ.

### Resilience to AD pathologies

Despite the increased prevalence of AD as we age, some individuals simply do not develop AD pathologies even into their 10^th^ or 11^th^ decade of life. Unless afflicted by other disorders these individuals show relatively preserved cognition [253]. Thus, AD is not an inevitable consequence of aging. Efforts are underway to understand why some individuals resist development of AD pathologies even far into advanced age –including those at genetic risk for the disease. These efforts are beginning to yield clues. Having protective genes certainly helps, but it is clear genetics is not the only factor [253–255]. In addition to ongoing efforts to identify factors that contribute to the development of AD, it seems that further investments to uncover both why some do not develop AD pathologies and why others resist functional decline in the face of these pathologies are warranted. Indeed, these observations offer hope that AD is not inevitable and that effective interventions can be identified.

## Conclusions: A future where AD is preventable, curable, and manageable

As the genetic underpinnings of AD were unraveled in the 1990s and druggable targets that modulated Aβ were identified, there was optimism that effective disease modifying therapeutics for AD would be rapidly developed, shown to work in humans, and then rapidly deployed. Unfortunately, this optimistic scenario did not play out. Instead, two decades of trying to target Aβ and now tau have yet to provide a transformative disease modifying therapy, despite the fact that many of these drugs showed disease modifying effects in preclinical models. There have been many published analyses of the reasons for these therapeutic trial failures [256–259]. In retrospect, it is clear that many drugs failed to engage to the appropriate target, in other cases, the drugs were too toxic, and in other cases, it was likely the drugs were not given at the appropriate time with respect to the disease progression.

Impressive efforts remain underway to address many aspects of the failed therapeutic pipeline for AD therapeutics. Among the most laudable of these are efforts to develop paradigms to study therapeutics in individuals with amyloid deposition before symptoms onset or even those at genetic risk without amyloid deposition [260–263]. These efforts remain a work in progress, and require therapeutics or other interventions that have a high degree of safety. Ultimately, such prevention studies can identify interventions that will have the most significant public health impact by delaying or preventing the development of AD in at risk individuals. Further, the amyloid- and tau-centric drug development approaches are being expanded to include numerous new targets within the brain immune, metabolic and vascular systems [259]. Finally, there are renewed efforts to find symptomatic therapies that not only target cognition but other aspects such as agitation that negatively affect patients with AD [264].

Clearly, having additional mechanistic insight into the complex cellular dysfunction that underlies the neurodegenerative phase of AD will help with these efforts [265]. Such an understanding should provide new targets and therapeutic strategies that might work at later stages of the AD degenerative cascade. Armed with better diagnostic tools, it is likely that we can in the future more precisely tailor any novel intervention in terms of selecting individuals most likely to benefit from that intervention. Clinical trials could be initiated at a time in AD pathogenic cascade, when the therapy would be most likely to show an efficacious disease modifying effect. Indeed, it has proven extremely hard to modify symptomatic AD by targeting amyloid deposition, even with therapeutics that appear to show good target engagement. This failure is not surprising given the triggering role of Aβ aggregate accumulation in AD. Such data simply inform us that once substantial neurodegenerative changes are present targeting the trigger is not sufficient to provide much benefit. The recent approval of aducanumab (Aduhelm) for AD by the FDA does little to alter the current therapeutic landscape for patients. Aducanumab appears to be an amyloid-clearing antibody, but its clinical impact is highly debated [266, 267]. Even though many are comfortable with the inference that lowering of the PET-amyloid ligand signal equates to actual reductions of amyloid plaques, the field lacks autopsy-confirmation that truly demonstrates plaque clearing by this antibody. As noted above, early symptomatic AD is not early from a pathological perspective, but rather the first signs of symptomatic brain organ failure. It is not surprising that amyloid-targeting therapies have little impact at this stage of disease [27].

I use the term brain organ failure to describe symptomatic AD to emphasize the fact that massive, longstanding and complex pathology is present in the brain even in the initial clinical stages of AD. Further, labeling symptomatic AD as brain organ failure provides a tangible, easily conveyed framework for understanding both why current trigger-targeting disease-modifying strategies have failed and for devising better therapeutic strategies in the future [79, 258]. Indeed, just as we treat heart failure with multiple drugs, we likely must target multiple aspects of AD’s underlying pathophysiology in order to significantly modify the disease course in symptomatic AD. Whether a single drug with pleiotropic actions or a combination of more targeted therapies can provide such efficacy is unknown, but a goal we must collectively pursue. Likely, these drugs will need to have some ability to restore or regenerate functional brain circuits in order to have truly transformative impacts. As developing even one effective disease modifying AD therapeutic has proven extraordinarily challenging, it is clear that developing combination therapies will be even more challenging.

Future efforts will likely find effective prophylactic interventions for AD [268]. Although AD prevention trials are challenging to conduct, it is likely that robust, safe interventions that either target Aβ, the unknown factors through which Aβ drives tau pathology, or tau induced neurodegeneration would prove efficacious in these studies. Until such prophylactic interventions are proven and optimized, millions more will still get AD. We must find more effective ways to help those currently suffering from AD and those at risk for developing AD. Even though we have not yet succeeded, I believe concerted multipronged efforts will lead to a future where AD is treated more effectively and ultimately becomes both preventable and curable.

## Data Availability

Not applicable.

## Abbreviations

Aβ: Amyloid β protein
ABI3: ABI family member 3
ACH: Amyloid cascade hypothesis
AD: Alzheimer’s Disease
APOE: Apolipoprotein E
APP: Amyloid β precursor protein
BACE1: β-Secretase
CAA: Congophillic amyloid angiopathy
CLU: Clusterin
CSF: Cerebrospinal Fluid
CNS: Central Nervous System
CTE: Chronic Traumatic Encephalopathy
DN: Dystrophic Neurite
EOAD: Early onset AD
EOFAD: Early onset familial AD
FBD: Familial British dementia
FDD: Familial Danish Dementia
FTD: Frontotemporal dementia
GFAP: Glial fibrillary acidic protein
GRN: Granulin
GWAS: Genome wide association study
HCHWA-D: Hereditary cerebral hemorrhage with amyloidosis – Dutch type
HSPG: Heparan sulfate proteoglycan
MAF: Minimum allele frequency
MAPT: Microtubule associated protein tau
MCI: Mild Cognitive impairment
MRI: Magnetic Resonance Imaging
NFL: Neurofilament light chain
NFT: Neurofilament heavy chain
PET: Positron emission tomography
PLCG2: Phospholipase C gamma 2
PSEN1: Presenilin 1
PSEN2: Presenilin 2
TBI: Traumatic brain injury
TDP-43: TAR DNA-binding protein
TM106b: Transmembrane protein 106b
TREM2: Triggering receptor expressed on myeloid cells 2
